# Case report: Bullous pemphigoid in HIV-1-positive patients: interplay or coincidence? A case series and review of the literature

**DOI:** 10.3389/fimmu.2023.1179294

**Published:** 2023-05-24

**Authors:** Yannick Foerster, Lukas Sollfrank, Laura Rechtien, Thomas Harrer, Carola Berking, Michael Sticherling

**Affiliations:** ^1^ Department of Dermatology, Universitätsklinikum Erlangen, Friedrich-Alexander-Universität Erlangen-Nürnberg, Erlangen, Germany; ^2^ Deutsches Zentrum Immuntherapie, Erlangen, Germany; ^3^ Infectious Diseases and Immunodeficiency Section, Department of Internal Medicine 3, Universitatsklinikum Erlangen, Erlangen, Germany

**Keywords:** bullous pemphigoid, HIV-1, aids, immune reconstitution inflammatory syndrome, iris, dupilumab, COVID-19

## Abstract

Bullous pemphigoid (BP) is an autoimmune inflammatory skin disease, mostly affecting the elderly population. Therefore, patients often have multiple comorbidities, but there is inconsistent data regarding the relationship between HIV-1 infection and BP, which has been rarely reported in combination. Herein, we describe three patients who presented with BP and concomitant HIV-1 infection that was well controlled with modern combined antiretroviral therapy. All patients received topical and oral corticosteroids. Depending on the individual severity, further add-on therapeutics, such as azathioprine, dapsone, doxycycline and the interleukin 4/13 antibody dupilumab, were added to the therapy regimen. All patients recovered from pruritic skin lesions and blistering. The cases are further discussed in the context of the current study landscape. In conclusion, HIV-1 infection shifts the cytokine profile from T-helper type 1 (TH1) towards T-helper type 2 (TH2), resulting in the excessive secretion of distinct cytokines, such as interleukin 4 (IL-4) and interleukin 10 (IL-10). With IL-4 being a main driver in the pathogenesis of BP, HIV-1-positive patients may benefit greatly from targeting IL-4 with monoclonal antibodies.

## 1 Introduction

Bullous pemphigoid (BP) is the most frequent autoimmune blistering disease with a prevalence of 0.13% in Europe, mostly affecting the elderly population (age > 60 years) (1). The disease is caused by autoantibodies against the hemidesmosomal proteins, BP180 and BP230, which are crucial for the attachment of epidermal cells to the basal lamina (2). In consequence, patients typically present with tense subepidermal blisters on erythematous skin accompanied by strong pruritus. However, clinical presentation is highly pleomorphic, ranging from eczema-like to multiform or urticarial lesions (3). Most cases occur spontaneously, but there are several trigger factors, such as drugs (oral antidiabetics, antibiotics, gold), human leukocyte antigen (HLA) polymorphisms (HLA-DQB1*0301), infections, vaccinations or malignant neoplasms that may induce or exacerbate BP (4). BP is also associated with different autoinflammatory diseases, and, among other factors, the TH1/TH2 and TH17/Treg homeostasis is considered to play a key role in the development of BP and other autoimmune diseases (5). Human immunodeficiency virus 1 (HIV-1) infection and acquired immunodeficiency syndrome (AIDS) lead to a T cell imbalance not only as a result of CD4+ T cell depletion, but also due to reconstitution of the immune system after initiation of a highly effective antiretroviral therapy (HAART) (6). Immune reconstitution inflammatory syndrome (IRIS) therefore contributes to the development of a variety of autoimmune disorders including Sjogren’s syndrome, psoriasis, systemic lupus erythematosus (SLE) and uveitis (6–8). However, there is inconsistent data regarding the association between HIV-1 infection, IRIS and BP. Herein, we describe three HIV-1-positive patients who presented with severe BP and we review the current literature to clarify the relationship between HIV-1 infection and BP.

## 2 Case presentation

### 2.1 Case 1

In October 2022, a 60-year-old man presented with a two-week history of pruritus, erythema and tender blisters on his body. Before admission, he had been treated with intravenous steroids and antihistamines once for suspected acute urticaria. However, symptoms worsened over the following days. The patient had a history of HIV-1 CDC A2 (classification system according to the Center for Disease Control and Prevention) infection, which had been diagnosed in 2004 and was well controlled with antiretroviral triple therapy (bictegravir 50 mg, emtricitabine 200 mg and tenofovir alafenamide 25 mg) once daily. Viral load was <20 copies/mL and CD4+ count was 418/μL. He had received a third dose of an mRNA-based COVID-19 vaccine in July 2022 and reported no prior history of COVID-19 infection.

**Figure 1:**
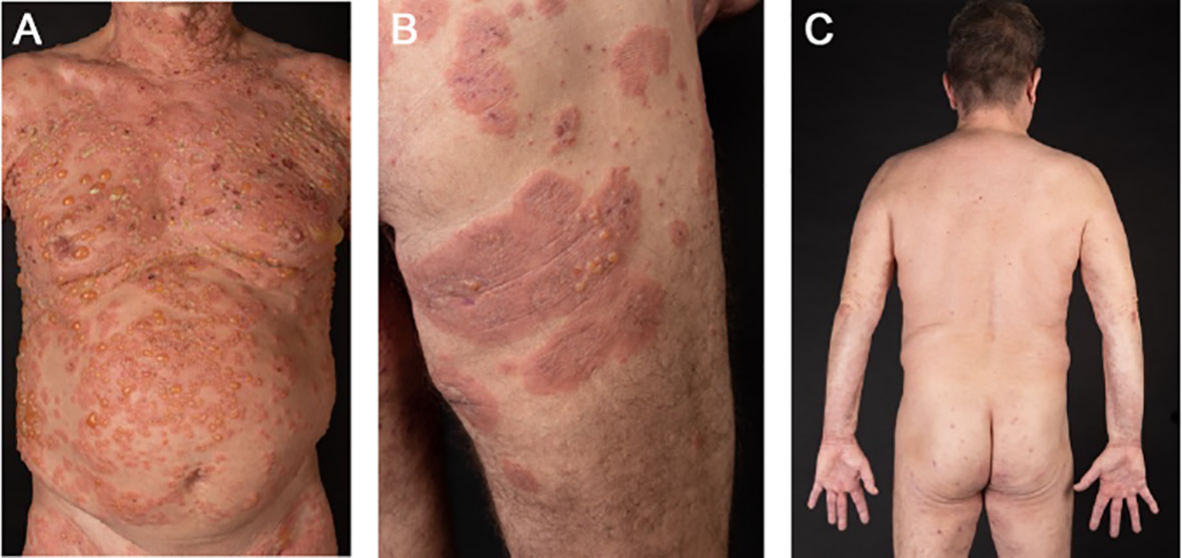
Case 1: In October 2023, a 60-year-old male presented to us with multiple blisters on erythematous skin **(A)**. A treatment with topical and oral steroids was administered plus intravenous immunoglobulins and dapsone as add-on therapy. Three weeks later, he presented again with pruritus, erythema and multiple bullae **(B)**, hence dupilumab was added to the treatment with dapsone and prednisolone. The skin continuously improved over the following weeks and oral steroids were tapered. At the 2-month follow-up, the patient did not show any new pruritic lesions or blistering **(C)**.

At physical examination, multiple blisters ranging in size from millimeters to several centimeters and crusted lesions on erythematous skin were found on his whole body with accentuation on his thighs and upper body (**Figure 1A**). Differential blood count revealed 11% (Ref. 2-4%) of eosinophilic granulocytes (1716/μL total). Total IgE level was 810 kU/L (Ref. <200 kU/L). However, the patient did not have a history of atopic dermatitis, asthma or chronic rhinitis. Highly elevated serum levels of anti-BP180 autoantibodies (147.7 U/mL, Ref. 0-9 U/mL) and linear IgG, IgM and C3 deposits at the dermal-epidermal junction (DEJ) confirmed the diagnosis of BP. For induction therapy, topical mometasone twice daily and oral therapy with prednisolone 80 mg (1 mg/kg body weight) once daily were administered. Oral doxycycline 100 mg twice daily was added to the initial therapy regimen and switched to dapsone up to 150 mg once daily after 7 days. After two weeks, the patient achieved partial remission, but still developed new lesions with blistering, so he additionally received a total of 160 g (2 g/kg body weight) of intravenous immunoglobulin (IVIG) over 4 days. During the following days no new blisters appeared and he was discharged from hospital after 18 days of treatment. Three weeks later the patient presented with a new flare of pruritic skin erosions and blistering (**Figure 1B**). At this time oral prednisolone was tapered (10-20 mg per week) to a dose of 30 mg once daily. Hence, dupilumab 600 mg was administered subcutaneously for induction and followed by 300 mg every two weeks for maintenance. Also, the dose of oral prednisolone was increased up to 80mg (1 mg/kg body weight) per day. He continued with dapsone 150 mg once daily and oral prednisolone was again tapered over the following weeks (10 mg per week) and then stopped completely due to significant improvement of the skin. At the 2-month follow-up visit in January 2023, the patient reported major relief with no new pruritic lesions or blistering (**Figure 1C**).

### 2.2 Case 2

A 62-year-old man, who had been diagnosed with HIV-1 CDC B3 in 2006, presented to us with pruritic urticarial lesions on his arms in February 2022. HIV-1 infection was well controlled with antiretroviral therapy (elvitegravir 150 mg, cobicistat 150 mg, emtricitabine 200mg and tenofovir alafenamide 10 mg). His viral load was <20 copies/mL and the CD4+ count 318/μL. In addition, he had a prior history of currently asymptomatic coronary heart disease, arterial hypertension, chronic kidney disease, hyperuricemia, steatosis hepatis, nicotine dependance (60 pack years) and alcohol abuse (3 liters of beer per day plus liquor). He also had a five-year history of hyperkeratotic papules on his right chest and the diagnosis of Darier’s disease was confirmed in 2021. Concomitant medications were acetyl salicylic acid, allopurinol, fluvastatin, ramipril, amlodipine, bisoprolol, folic acid and torasemide. He had a history of mild COVID-19 infection in May 2021 and received his second dose of an mRNA-based COVID-19 vaccine in December 2021. Biopsy revealed subepidermal blistering and eosinophilic infiltration accompanied by linear deposition of IgG and C3 at the DEJ. Anti-BP180 autoantibodies were 1735.4 U/mL. Upon hospitalization, topical mometasone twice daily, oral prednisolone 60 mg (0,5 mg/kg body weight) and dapsone 50 mg once daily were initiated. In clinical remission the patient was discharged from hospital after 9 days. Under treatment with oral dapsone over the following 6 weeks, hemoglobin concentration (Hb) fell from 14,6 g/dL (Ref. 13,5-17,5 g/dL) to 12,2 g/dL. Methemoglobin level also increased from 1,1% (Ref. <1%) to 3,2% and the patient complained about progressive dyspnea. Therefore, dapsone had to be discontinued, which resulted in a relapse of pruritic urticarial lesions and blisters. Consecutively, the patient received azathioprine 100 mg/day and prednisolone was tapered over the following weeks (10mg per week). As the patient developed pulmonary embolism, he received anticoagulation with apixaban and his antiviral medication was switched from elvitegravir/cobicistat to bictegravir, also in combination with emtricitabine and tenofovir alafenamide. The cutaneous lesions gradually improved over the following months, formation of new blisters ceased and erythema faded. After six months of treatment azathioprine had to be stopped in December 2022 because of pancytopenia (Hb: 10 g/dL, Ref. 13,5 – 17,5 g/dL; Leukocytes: 2990/μL, Ref. 4000-10000/μL; Thrombocytes 127000/μL, Ref. 150000-300000/μL) and general malaise. So far the patient did not develop any new pruritic lesions or bullae off-therapy, though anti-BP180 autoantibodies were still detectable at a concentration of 241.8 U/mL. However, since Darier’s disease severely deteriorated with plaque formation, acitretin 10 mg once daily was started in January 2023.

### 2.3 Case 3

In August 2021, a 74-year-old female presented with a three-week history of pruritic exanthema and flaccid blisters on her abdomen and both arms. She had been diagnosed with HIV-1 CDC C3 in 2003, which was well controlled with emtricitabine 200 mg, tenofovir disoproxil 245 mg and efavirenz 600 mg once daily. At the time of skin disease, viral load was 20 copies/mL and CD4+ count was 383/μL. She had a history of chronic hepatitis C (diagnosed 2003) which had successfully been treated with ribavirin and pegylated interferon-alpha in 2006, coronary heart disease with coronary artery bypass graft (ACBG), biological aortic valve replacement, mitral valve repair, chronic obstructive pulmonary disease (COPD), first-degree atrioventricular block and nicotine abuse (30 packyears). The patient had no history of COVID-19 infection or vaccination and no COVID-19 antibodies were detectable. Direct immunofluorescence showed linear C3 deposits at the DEJ. Elevated levels of anti-BP180 (392.2 U/mL) and anti-BP230 (49.9 U/mL) autoantibodies confirmed the diagnosis of a BP. When topical treatment with mometasone twice daily was administered with only partial response and little impact on pruritus, oral prednisolone with 30 mg daily per os (0,5 mg/kg body weight) was added. To achieve full disease control, the patient was given doxycycline 200 mg once daily as add-on therapy. Under treatment with oral doxycycline the patient did well without any recurrent lesions and she was discharged from hospital after seven days. The patient did not attend to a follow-up visit. Overview of cases 1-3 are given in **Table 1**.

**Table 1:** Overview of cases.

Case	Age	Gender	HIV type	HIV-1 diagnosis	CDC stage	HIV-1 treatment	BP diagnosis	anti-BP180 [U/mL]	Therapy	COVID-19 disease	COVID-19 vaccination
1	60	male	HIV-1	2004	A2	emtricitabine, tenofovir-alafenamide, bictegravir	Oct 22	147.7	Topical mometasone, prednisolone, doxycycline, IVIG, dupilumab		3rd in July 2022 (mRNA based)
2	62	male	HIV-1	2006	B3	emtricitabine, tenofovir-alafenamide, elvitegravir, cobicistat (7/22 evg/c switched to bictegravir)	Feb 22	1735.4	Topical mometasone, prednisolone, dapsone, azathioprine	May 2021	2nd in December 2021 (mRNA based)
3	74	female	HIV-1	2003	C3	emtricitabine, tenofovir-disoproxil, efavirenz	Aug 21	392.2	Topical mometasone, prednisolone, doxycycline		

HIV, human immunodeficiency virus; CDC, Center for Disease Control and Prevention; BP, bullous pemphigoid; evg, elvitegravir; c, cobicistat.

## 3 Discussion

The relationship between dysregulation of the immune system and HIV-1 infection is complex and has not yet been fully understood. Until now, almost 40 years have elapsed since the onset of the HIV-1 epidemic in the mid-1980s (9). Since the introduction of antiretroviral therapy, the disease has changed from a life-limiting disorder to a chronic one, leading to the arise of dominant comorbidities, such as autoimmune diseases or sarcoidosis (6). With regard to the three cases presented here, the following scenarios of HIV-1-BP relation may be discussed: 1. An increased rate of BP autoantibodies in HIV-1 infection, 2. A modulation of BP autoantibodies by HAART, 3. A distinct role of IL-4 in both diseases and 4. The impact of COVID-19 or vaccination.

There are various autoantibodies that are detected more or less frequently in HIV-1-infected people than in HIV-1-negative controls, but only limited data exist on HIV-1 infection and BP autoantibodies anti-BP180 and anti-BP230. A study by Touzeau-Roemer et al. showed comparably low prevalence of anti-BP180 and anti-BP230 autoantibodies in HIV-1-infected patients and HIV-1-negative controls with 6.23% (anti-BP180) and 5.72% (anti-BP230) of 594 HIV-infected patients, respectively, compared with 5.24% (anti-BP180) and 4.03% (anti-BP230) of 248 negative controls (10). Another study of the pre-HAART era by Kinloch-de Loës et al. revealed that the incidence of BP autoantibodies is significantly higher in HIV-1-positive than in HIV-1-negative persons (11). Within this group, circulating BP autoantibodies were found in the serum of 38% of 90 HIV-1-infected patients compared to only 21% in the serum of 21 negative controls suffering from chronic pruritus. The frequency was also dependent on the duration of the HIV-1 infection, increasing from 21% in early disease to 40% in advanced stages (11). To our knowledge, only three case reports of patients with both HIV-1 infection and BP have been published so far (**Table 2**). In line with the findings of the previous studies, two case reports are from the pre-HAART era (12–14). These findings suggest that subsequent reconstitution of the immune system by HAART may reduce the risk of developing autoantibodies. Accordingly, recent studies have shown that there is an inverse correlation between the presence of antinuclear antibodies (ANA), anti-extractable nuclear antigens (anti-ENA), antineutrophil cytoplasmic antibodies (ANCA) and CD4+ count due to successful HAART (15).

**Table 2:** Published cases of patients with bullous pemphigoid (BP) and concomitant infection with human immunodeficiency virus (HIV).

Case	Published	Gender	Age	HIV diagnosis	HIV treatment	BP diagnosis	Clinical findings	Diagnostic findings	Therapy	Outcome
Levy et al. (12)	1986	male	58	1983	none	Nov 84	pruritus, erythematous papules	eosinophilia (16%), linear IgG at DEJ	ritodrine	pruritus completely resolved after 4 weeks, promptly returned after discontinuation of ritodrine
Bull et al. (13)	1993	male	58	1985	none	Sep 87	tense blisters on legs	histological examination with subepidermal blistering, linear IgG at DEJ	oral prednisolone	died due to pneumocystis pneumonia in 1990
De et al. (14)	2008	male	30	year NA, HIV diagnosis 10 years before onset of BP	nevirapine, lamivudine, stavudine (started 6 weeks before onset of BP)	NA	pruritus, tense blisters, erosions	histological examination with subepidermal blistering and eosinophilic infiltration	oral prednisolone	all lesions completely disappeared and prednisolone was tapered

There is no information available regarding the HIV subtypes. NA, not available; IgG, immunoglobulin G; DEJ, dermal-epidermal junction.

On the other hand, successful antiretroviral treatment may lead to alterations of the T cell balance with excessive immune response and development of autoimmune disorders. For example, in patients with chronic HIV-1 infection increased levels of FoxP3-positive regulatory T cells (Tregs) are found, which upon successful treatment of the viral infection leads to transformation of the Tregs into interleukin 17 (IL-17)-producing T cells. These may in turn induce novel or exacerbate a pre-existing psoriasis (16–18).

Besides immunoglobulin G (IgG)-induced inflammation at the epidermal basement membrane, almost 50% of patients with BP have elevated serum levels of immunoglobulin E (IgE) and blood eosinophilia. This indicates that a T-helper type 2 (TH2)-polarized autoimmune response plays an important role in the pathogenesis of BP (19). It is known that during the course of HIV-1 infection secretion of TH1 cytokines, such as interleukin 2 (IL-2), and interferon γ (IFNγ), is generally decreased, whereas production of TH2 cytokines, interleukin 4 (IL-4), interleukin 10 (IL-10) and interleukin 13 (IL-13), is increased (20, 21). This observation suggests that TH2-mediated immune response with excessive release of IL-4, IL-10 and IL-13 may be the predominant pathomechanism in HIV-1-positive patients with BP. This may also explain why BP autoantibodies increase from early to advanced stages of the HIV-1 infection as seen in the study by Kinloch-de Loës et al. A successful antiretroviral therapy counteracts this TH1/TH2 and cytokine shift, thus reducing the seroprevalence of circulating anti-BP180 and anti-BP230 autoantibodies in HIV-1-positive patients who receive HAART. However, there is evidence that serum levels of TH2 cytokines decrease after initiation of HAART, but still persist at higher levels compared to HIV-1 negative controls (22).

If IL-4/IL-13 is assumed to be the main driver of BP especially in HIV-1-positive patients, it may also explain why patient 1 responded so well to the treatment with the monoclonal IL-4/IL13 antibody dupilumab. Dupilumab as a possible treatment option for patients suffering from moderate to severe BP has also been described in few case reports and case series (23–25) and is currently evaluated in ongoing controlled clinical studies.

Remarkably, three HIV-1-positive patients with BP presented to our department in 2021 and 2022 whereas over a long time before, no single patient has been seen. Two patients developed blistering in temporal relationship to mRNA-based vaccination against COVID-19, and there is an ongoing discussion that SARS-CoV-2 vaccines might play a role in BP initiation in healthy adults (26). The induction of new or exacerbation of existing BP by COVID-19 or respective vaccination is currently controversially debated, yet available data do not support a clear relation different from other viral infections. As known for different chronic inflammatory diseases, an induction or exacerbation may be anticipated in patients with appropriate immunogenetic background. In fact, a large population-based study by Birabaharan et al. evaluated available health data of over 70 million people who had received mRNA-based vaccines between December 2020 and June 2021. No difference in risk of new-onset BP was seen among patients who had received mRNA COVID-19 vaccine compared to the control cohort (27). However, recent studies report the development of immune reconstitution inflammatory syndrome (IRIS) in HIV-1-positive patients following COVID-19 infection due to dysregulated innate immune responses generated in the absence of effective adaptive immune responses (28). Stevenson et al. recently reported that the COVID-19 mRNA vaccine BNT162b2 leads to transcription of HIV proviruses with consecutively enhanced T-cell induction (29). This in turn may also affect autoimmunity, suggesting mRNA vaccination may act as a cofactor in the pathogenesis of autoimmune disorders in HIV-positive patients. In conclusion, the risk for BP may increase over the course of HIV-1 infection either following disbalance of T-cell responses or due to enhanced TH2-mediated immune response, leading to altered cytokine profiles with high levels of IL-4 and IL-10. Targeting of IL-4 and IL-10 by monoclonal antibodies may therefore be very effective for patients suffering from both HIV-1 and BP. However, due to the limited number of cases and published data, further prospective studies must evaluate serum levels of BP autoantibodies over the course of HIV-1 infection. Randomized controlled trials evaluating dupilumab as a treatment for BP should also focus on patients with concomitant HIV-1 infection to prove its effectiveness for this special patient group.

## Data availability statement

The original contributions presented in the study are included in the article/supplementary material. Further inquiries can be directed to the corresponding author.

## Ethics statement

Written informed consent was obtained from the individual(s) for the publication of any identifiable images or data included in this article.

## Author contributions

YF, LS, LR, TH, CB and MS participated in the examination and treatment of one or more of the patients. YF and MS collected the data and wrote the manuscript. CB, TH, LS and LR reviewed the manuscript and aided with language prettification. All authors contributed to the article and approved the submitted version.
